# Effects of Steviol Glycosides on Growth Performance, Ruminal Fermentation and Microbial Diversity of Hu Sheep

**DOI:** 10.3390/ani14131991

**Published:** 2024-07-05

**Authors:** Jianeng Zhang, Xiongxiong Li, Yuzhu Sha, Zhengwen Wang, Shuai Qi, Xia Zhang, Shengguo Zhao, Ting Jiao

**Affiliations:** 1 College of Pratacultural Science, Gansu Agricultural University, Lanzhou 730070, China; zhangjn@st.gsau.edu.cn (J.Z.); gsauwangzhengwen@163.com (Z.W.); idreamworker@sina.com (S.Q.); 18894012309@139.com (X.Z.); 2 Key Laboratory for Grassland Ecosystem of Ministry of Education, Sino-US Grassland Animal Husbandry Sustainable Development Research Center, Gansu Agricultural University, Lanzhou 730070, China; 3 College of Animal Science and Technology, Gansu Agricultural University, Lanzhou 730070, China; lxx_gsau@163.com (X.L.); 18894314093@163.com (Y.S.); zhaosg@gsau.edu.cn (S.Z.)

**Keywords:** steviol glycosides, Hu sheep, rumen fermentation, volatile fatty acids, microbial diversity

## Abstract

**Simple Summary:**

Long-term use of feed additives containing antibiotics and chemicals can make some pathogens resistant and even cause mutations that threaten animal and human health. Therefore, the development of green feed additives has become a top priority. Recent studies have shown that steviol glycosides are helpful in improving livestock performance, improving feed utilization, and regulating gastrointestinal microbiota. At present, steviol glycosides have been used as feed additive in pigs, fish and birds, but less in ruminants. Therefore, the primary aim of this study was to delve into the impact of steviol glycosides on the performance, blood biochemical parameters, rumen fermentation processes, and the microflora composition of Hu sheep. The findings revealed that the inclusion of steviol glycosides did not exert any notable influence on the growth rate or serum biochemical indices of Hu sheep, but it could improve rumen fermentation parameters and the structure of the rumen microbial community, had certain effects on the diversity and composition of rumen microorganisms, and helped maintain the balance of rumen microecology.

**Abstract:**

The experiment was conducted to investigate the potential effects of steviol glycosides on growth performance, rumen fermentation processes, and microbial diversity in Hu sheep. A single-factor design was used for the trial. Twenty healthy weaned Hu lambs, possessing comparable body weights averaging 18.31 ± 1.24 kg, were randomly allocated into two distinct groups: the control group (CON) and the experimental group (STE), with each comprising 10 lambs. The CON was fed the basal diet, and the STE was supplemented with 0.07% steviol glycosides based on the basal diet. During the experimental period, variations in body weight and feed intake were closely monitored and recorded. After feeding for 90 d, blood was collected to determine blood biochemical indices, and rumen fluid samples were gathered for an in-depth analysis of rumen fermentation parameters and microbial diversity. The outcomes revealed no statistically significant differences in growth performance or serum biochemical indices between the two groups (*p* > 0.05). Rumen pH in STE and CON was within the normal range. The rumen ammonia nitrogen (NH_3_-N) and acetic acid (AA) content of STE decreased significantly compared with CON (*p* < 0.05). No significant variations were observed in the levels of other volatile fatty acids (VFAs) between the two groups (*p* > 0.05). The rumen microbial OTUs count, as well as the Shannon, Simpson, Chao1, and Ace indices, were notably lower in the STE group compared to the CON group (*p* < 0.05). Additionally, at the phylum level, the relative abundance of *Firmicutes*, *Bacteroidetes*, and *Proteobacteria* collectively accounted for over 97% of the total phylum composition. In comparison to the CON group, the STE group exhibited an increase in the relative abundance of *Proteobacteria* (*p* < 0.05), accompanied by a significant reduction in the relative abundance of *Patescibacteria* and *Desulfobacteria* (*p* < 0.05). At the genus level, there was a notable increase in the relative abundance of *Prevotella_7* and *Succinivibrionaceae_UCG_001* in the STE group, whereas the relative abundance of *Rikenellaceae_RC9_gut_group* significantly decreased (*p* < 0.05). According to the correlation analysis between rumen microflora and VFAs, the relative abundance of *Succinivibrionaceae_UCG_001* displayed a significant negative correlation with AA (*p* < 0.05), whereas *Lactobacillus* exhibited a notable positive correlation with isobutyric acid (IBA) (*p* < 0.05). In summary, steviol glycosides had no significant effect on the production performance and blood biochemical indexes of Hu sheep. Steviol glycosides can improve rumen fermentation parameters and rumen microflora structure of Hu sheep and have a certain effect on rumen microbial diversity and composition.

## 1. Introduction

In recent years, China has witnessed remarkable advancements in the scale and industrialization of animal husbandry, and the output of livestock products has grown steadily, making an important contribution to the national economy. However, it should also be noted that with the rapid development of animal husbandry, some problems have gradually become prominent [1]. Long-term use of feed additives containing antibiotics and chemicals will cause some pathogens to develop resistance and even cause mutations, threatening animal and human health [2]. Therefore, the development of green feed additives has become a top priority. Feed additives can not only improve the nutritional value of feed and optimize the internal environment of the rumen but also enhance or restrict the structure of specific microbial flora, regulate the microecological balance of the animal intestine, and enhance immunity [3].

As a natural sweetener, steviol glycosides have received much attention due to their unique biological characteristics and potential benefits to animal health [4]. Steviol glycosides, also known as stevia, is a natural diterpenoid glycoside extracted from stevia, a perennial herb in the *chrysanthemum* family. It has functions of lowering blood pressure, inhibiting bacteria, and enhancing immunity [5]. Stevia was widely planted in many provinces and cities in China, so China is the most important producer of steviol glycosides [6]. Recent studies have revealed that steviol glycosides effectively enhance the performance of livestock and poultry, boost feed utilization, and regulate the gastrointestinal microbiota. [7]. Research has confirmed that incorporating stevia residue into the diet of pregnant sows effectively modulates the composition of intestinal flora, leading to a significant increase in the relative abundance of intestinal beneficial bacteria [8]. The inclusion of steviol glycosides in the diet of mirror carp has been shown to enhance growth performance, liver antioxidant capacity, and immune function [9]. The addition of steviol glycosides could improve feed intake and the apparent digestibility of neutral and acid-detergent fibers of goats [10]. It has also been found that dietary steviol glycosides can improve the daily egg production and feed conversion rate of aged laying hens [11].

Rumen is an important part of the digestive system of ruminants, and its fermentation process directly affects the digestibility of feed and the health of animals [12]. The microorganisms in the rumen not only participate in feed decomposition and transformation but also have a profound impact on sheep nutrient absorption and health status [13]. At present, there are extensive studies on steviol glycosides as a feed additive in pigs, fish, and birds, but there are few studies on ruminants. Therefore, we conducted this study to explore the effects of steviol glycosides on production performance, blood biochemical indexes, rumen fermentation, and microflora of Hu sheep, and the aim is to provide a theoretical basis for the application of steviol glycosides in Hu sheep production practices, thereby optimizing and guiding these practices for better outcomes.

## 2. Materials and Methods

### 2.1. Experimental Design

The experiment was designed by a single factor. Twenty 1.5-month-old male Hu lambs with similar body weight (18.31 ± 1.24 kg) and healthy body condition were selected from Jinheyuan Ecological Family Farm (103° E, 35° N), Guanghe County, Gansu Province. The lambs were randomly assigned to two groups, namely the control group (CON) and the experimental group (STE), with 10 lambs in each group. Lambs of the CON were fed the basal diet, and the STE was supplemented with 0.07% steviol glycosides (based on air drying) based on the basal diet. The added amount of 0.07% steviol glycosides was selected based on our previous study [14]. The trial lasted for 90 d, including a 15 d adaptation period and 75 d of data collection. Feed intake was measured daily, and the weight of Hu sheep was measured every 15 days. At the end of the experiment, blood was collected for blood biochemical index determination, and rumen contents were collected for rumen fermentation determination and microbial analysis from six Hu sheep in each group randomly.

### 2.2. Basic Diet and Nutrition Level

The basal diet was formulated in accordance with the Chinese meat sheep feeding standard (NY/T 816-2004) [15] and comprised a ratio of 7:3 whole silage corn to concentrate supplement. The composition and nutritional levels of the concentrate supplement are detailed in Table 1.

### 2.3. Feeding and Management

Before the experiment, the sheepfold was thoroughly cleaned and sanitized, including the sheep pens, feeding troughs, and other facilities. The sheep were fed twice daily (8:00 a.m. and 5:00 p.m.), with free access to water in between. The sheepfold was regularly cleaned to maintain cleanliness and regular immunization was conducted in accordance with the requirements of the breeding farm.

### 2.4. Sample Collection and Processing

In order to reduce the pain and discomfort of animals, we tried to reduce the number of sheep that needed blood collection. Therefore, after the experiment, six Hu sheep in each group were randomly selected for jugular vein blood collection, and the blood collection volume was 15 mL. Following a two-hour storage period, the blood samples were centrifuged at 3500 r.min^−1^ for 15 min. The resulting fresh serum was collected and stored in a refrigerator at −20 °C for future use. Subsequently, the sheep were slaughtered. The rumen was quickly removed from the abdominal cavity. Approximately 200 mL of rumen fluid was collected and filtered through 4 layers of gauze. The filtered rumen fluid was subsequently transferred into a 450 mL centrifuge tube. The centrifuge tube was quickly frozen in a liquid nitrogen tank. After being brought back to the laboratory, they were transferred to the refrigerator at −80 °C for storage, and rumen fermentation parameters and microbial flora were determined.

### 2.5. Indexes and Measurement Methods

#### 2.5.1. Measurement of Production Performance Index

During the experiment, the initial weight and final weight of each Hu sheep were recorded, and the total dietary consumption, ADG, ADFI and F/G of each Hu sheep were measured during the experiment. The calculation formula is as follows [16]:

ADG/(kg/d) = Final weight − Initial weight/total test days;

ADFI/g = total dietary consumption during the experiment/total test days;

Feed to meat ratio (F/G) = ADFI/ADG.

#### 2.5.2. Determination of Serum Biochemical Indexes

The desired serum biochemical indices were analyzed using an automatic biochemical analyzer. Prior to analysis, the serum samples were thawed on ice and centrifuged at 3000× *g* for 10 min at 4 °C. Machine test according to kit instructions. The kits were purchased from Shanghai Peisenor Gene Technology Co., Ltd. (Shanghai, China) [16].

#### 2.5.3. Rumen Fermentation Index Determination

The pH of rumen fluid was measured using a pHS-10 portable pH meter. Ammonia nitrogen in rumen fluid was determined by the colorimetric method as referenced by Feng Zongci et al. [17]. The volatile fatty acid (VFA) content was measured using an Agilent 6890N (Manufactured by Agilent Technologies Inc., Santa Clara, CA, USA, and purchased in Shanghai, China) gas chromatograph. Sample preparation involved centrifuging rumen fluid at 5400 rpm for 10 min, followed by taking 1 mL of the supernatant and placing it in a 5 mL centrifuge tube. The solution was thoroughly mixed and kept in an ice-water bath for over 30 min before being centrifuged at 10,000 rpm for another 10 min. Parameters for the gas chromatograph inlet sample included carrier gas N_2_, split ratio of 40:1, injection volume of 0.6 μL, and temperature set to be at 220 °C. The chromatographic column operated in constant flow mode with a flow rate of 2.0 mL/min, average linear velocity of approximately 38 cm/s, and column pressure maintained at 11.3 psi. The detector settings were FID with a temperature set to 250 °C, H_2_ flow rate of 40 mL/min, air flow rate of 450 mL/min, combined column flow rate, and make-up gas flow rate of 45 mL/min. The oven temperature program ramped up from 120 °C (3 min) to 180 °C (1 min) at a rate of 10 °C/min [18].

#### 2.5.4. Ruminal Microbial Determination

Total DNA was extracted from the rumen microbial communities, following the detailed instructions provided by the Bacterial DNA Extraction Kit from Omega (Shanghai, China). DNA quality and concentration were detected by agarose gel electrophoresis and NanoDrop 2000 (Manufactured by Thermo Fisher Scientific, Waltham, MA, USA, and purchased in Shanghai, China), respectively. Specific primers were utilized to amplify the V3–V4 variable region of the 16S rRNA gene via polymerase chain reaction (PCR), aiming to capture the distinct characteristics of the rumen microflora. The amplification products underwent sequencing using the Illumina MiSeq platform (Illumina, San Diego, CA, USA). At a 97% similarity threshold, the effective sequences were clustered to generate operational taxonomic units (OTUs). These OTUs were subsequently annotated using the Silva (Bacteria) taxonomic database. Based on OTU analysis, species taxonomic analysis was carried out to obtain the number and species distribution histogram of each sample at each taxonomic level (phylum and genus level). The dilution curve and Alpha diversity index were obtained by analyzing the species diversity within a single Alpha sample. The PCoA map was obtained by analyzing the species diversity (composition and structure of flora) of different Beta samples. By LEfSe analysis, the histogram of LDA value distribution was obtained to find the different bacteria between the groups [18].

### 2.6. Statistical Analysis

Test data were preliminarily sorted out by Excel, and then the independent sample *t*-test in SPSS 26.0 software was used to analyze the significant differences. Analysis results were expressed as ‘mean ± standard deviation’. *p* < 0.05 indicates a significant difference. *p* > 0.05 indicated that the difference was not significant.

## 3. Results

### 3.1. Effect of Steviol Glycosides on Growth Performance of Hu Sheep

There was no significant difference in initial weight between STE and CON (*p* > 0.05). There were no significant differences in Net gain, ADG, ADFI, and F/G between the two groups (*p* > 0.05) (Table 2).

### 3.2. Effect of Steviol Glycosides on Serum Biochemical Indices of Hu Sheep

The dietary addition of steviol glycosides had no significant effect on the serum biochemical indices of Hu sheep in the two groups (*p* > 0.05) (Table 3).

### 3.3. Effect of Steviol Glycosides on Hu Sheep Rumen Fermentation

The pH values between CON and STE showed no significant variation (*p* < 0.05). However, a marked reduction was observed in the content of NH_3_-N and AA in the STE compared to the CON (*p* < 0.05). Conversely, there were no significant differences in the levels of propionic acid (PA), IBA, butyric acid (BA), isovaleric acid (IVA), valeric acid (VA), total volatile fatty acid (TVFA), and the AA to PA ratio (A/P) between the two groups (*p* > 0.05) (Table 4).

### 3.4. Effect of Steviol Glycosides on Hu Sheep Rumen Microorganism

#### 3.4.1. Alpha Diversity Analysis

**Figure 1:**
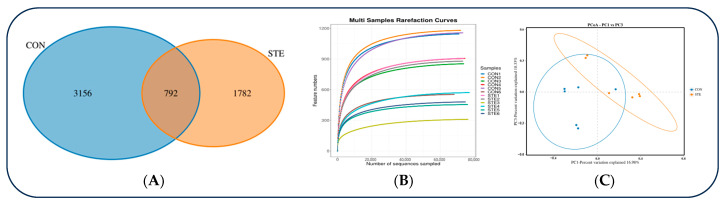
(**A**) Comparison of the differences in Venn diagrams between the two groups. (**B**) The differences comparison in dilution curves between the two groups. (**C**) Comparison of the differences in PCoA plots between the two groups.

A total of 960,991 original sequences were obtained by sequencing the 16S rRNA V3~V4 region of 12 samples in two groups in this experiment, of which 959,109 were valid sequences, and the sequence coverage was 99.8%. The Alpha diversity index of the samples was evaluated using QllME2 software (https://magic.novogene.com, accessed on 2 July 2024). It can be seen that a total of 5730 OTUs was obtained from samples of two groups at the 97% species similarity level, of which 792 were shared by two groups, accounting for 13.8% of the total number of OTUs. There were 3156 unique OTUs in STE and 1782 unique OTUs in CON, accounting for 55.08% and 31.10% of the total OTUs, respectively (Figure 1A).

The dilution curve basically tends to be gentle, indicating that the sequencing depth is reliable, which can truly reflect the composition of most microorganisms in samples and can be used for microbial diversity analysis (Figure 1B).

It can be seen that the rumen microorganisms Shannon–Wiener, Simpson, Ace, and chao1 indices of the CON were significantly higher than those of the STE (*p* < 0.05) (Table 5).

#### 3.4.2. Beta Diversity Analysis

The PCoA cluster analysis for rumen fluid bacterial OTU in the two groups is shown in Figure 1C. It can be seen that there are differences in rumen microorganisms between the two groups.

#### 3.4.3. Effect of Dietary Steviol Glycosides on Rumen Bacterial Taxonomic Composition and Community Structure (Phylum Level) on Hu Sheep

**Figure 2:**
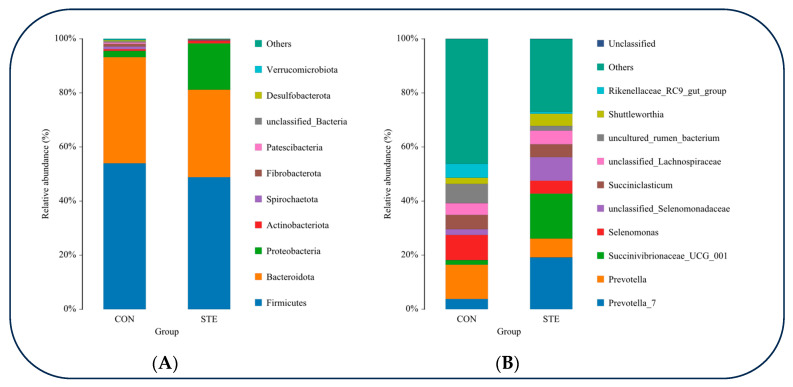
(**A**) Relative abundance of rumen microorganisms at gate level (**B**) Relative abundance of rumen microorganisms at genus level.

At the phylum classification level, a tally was conducted to determine the top 10 species exhibiting the highest relative abundance in the rumen microbial communities of the two groups. Among these, *Firmicutes* displayed the highest relative abundance in both groups, closely followed by *Bacteroidota*. The relative abundance of *Proteobacteria* in STE was significantly higher than that in CON (*p* < 0.05). The relative abundance of *Patescibacteria* and *Desulfobacterota* in STE was significantly lower than that in CON (*p* < 0.05) (Figure 2A and Table 6).

#### 3.4.4. Effect of Dietary Steviol Glycosides on Rumen Bacterial Taxonomic Composition and Community Structure (Genus Level) on Hu Sheep

At the genus classification level, we determined the top 10 species with the greatest relative abundance in the rumen microbial communities of the CON and STE. Among these, *Prevotella* exhibited the highest relative abundance in both groups. Notably, the relative abundance of *Prevotella_7* and *Succinivibrionaceae_UCG_001* was significantly greater in the STE compared to the CON (*p* < 0.05). Conversely, the relative abundance of uncultured_rumen_bacterium and *Rikenellaceae_RC9*_gut_group was significantly lower in the STE than in the CON (*p* < 0.05) (Figure 2B and Table 7).

#### 3.4.5. Analysis on Significant Differences of Rumen Flora

**Figure 3:**
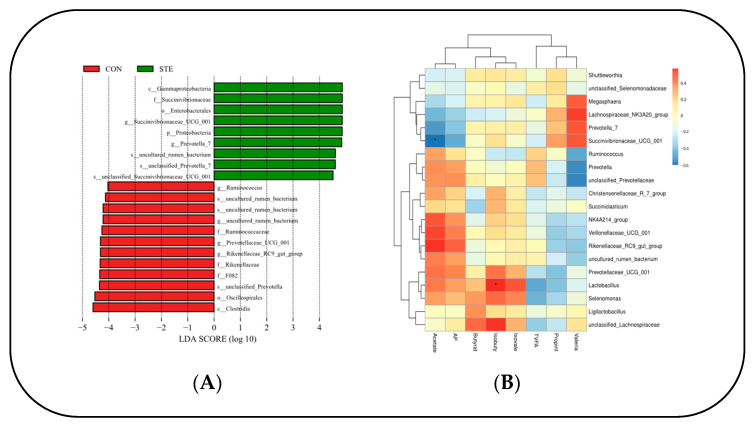
(**A**) Histogram of the distribution of LDA values. (**B**) Heat map of correlation between rumen microbial flora and VFAs in Hu sheep. * *p* < 0.05.

Through LEfSe analysis of the samples between the two groups, a histogram of LDA value distribution was obtained, showing significant differences in abundance between the two groups. It can be seen that there were 10 species groups with significant differences in the CON. *Clostridia*, *Oscillospirales*, *F082*, unclassified *Prevotella*, *Rikenellaceae*, *Rikenellaceae RC9* gut group, *Prevotellaceae_UGG_001*, *Ruminococcaceae*, uncultured rumen bacterium, and *Ruminococcus*. There were nine significantly different species in the STE, mainly *Gammaproteobacteria*, *Succinivibronaceae*, *Enterobacterales*, and *Succinivibronaceae_UGG_001* (Figure 3A).

#### 3.4.6. Correlation Analysis between Rumen Microbial Flora and VFAs

A correlation heat map was generated to illustrate the relationship between the top 20 genus-level microorganisms of the rumen microbial flora and volatile fatty acids (VFAs) in Hu sheep. The analysis revealed a significant negative correlation between *Succinivibrionaceae*_UCG_001 and AA in the rumen (*p* < 0.05). Conversely, *Lactobacillus* displayed a significant positive correlation with IBA in the rumen (*p* < 0.05). (Figure 3B).

## 4. Discussion

### 4.1. Effect of Steviol Glycosides on Growth Performance of Hu Sheep

The similarity of initial weights ensured that the two groups of Hu sheep were comparable at the beginning of the experiment. The similarity of final weight and the data of net weight gain and average daily weight gain further indicated that steviol glycosides supplementation did not significantly affect the growth rate of Hu sheep. This may be related to the higher sweetness but lower energy value of steviol glycosides. Although it can be used as a sweetener, it may not be significant in providing energy for growth [19]. For ADFI, there was no significant difference in ADFI between STE and CON (*p* > 0.05). This indicates that the addition of steviol glycosides may not significantly affect the appetite or feeding behavior of Hu sheep. The feed conversion rate (F/G) data further verified this point, indicating that the addition of steviol glycosides did not significantly affect the feed utilization efficiency [16]. As a natural sweetener, steviol glycosides are much sweeter than sucrose and have very low calories, so they may have potential applications in weight control and energy intake reduction [8].

### 4.2. Effect of Steviol Glycosides on Serum Biochemical Indexes of Hu Sheep

There were no significant changes in ALT and AST after steviol glycosides supplementation (*p* > 0.05). ALT and AST are important indicators of liver function, and the maintenance of their normal levels indicates that liver cells are not significantly damaged [20]. The results showed that the addition of steviol glycosides had no negative effect on the liver function of Hu sheep. The levels of TP and ALB decreased slightly after steviol glycosides supplementation (*p* > 0.05), which may be related to changes in protein sources in feed or the animals’ adaptive response to steviol glycosides [21]. However, since the difference is not significant, this change may not have a significant impact on Hu sheep’s overall health status. In terms of lipid indexes, there were no significant effects on the lipid metabolism of Hu sheep. HDL levels decreased slightly, while low-density LD levels increased slightly (*p* > 0.05), which may be related to the potential effects of steviol glycosides on lipid metabolism, but further studies are needed to be confirmed [22]. BUN increased, and GLU decreased slightly after steviol glycosides supplementation (*p* > 0.05). The increase in BUN level may be related to the increase in protein metabolism, while the decrease in GLU level may be related to the higher sweetness of steviol glycosides, resulting in animals’ other carbohydrate intake being reduced [23]. However, these changes were within the normal range, so it is unlikely to have a significant impact on the health of the Hu sheep. In summary, the addition of steviol glycosides had no significant effect on the serum biochemical indexes of Hu sheep. This result is consistent with some previous studies showing that the addition of steviol glycosides as a natural sweetener in animal feed generally does not negatively affect the health of animals [19]. The specific effects of steviol glycosides on animal biochemical indices may be influenced by many factors, such as additive amount, feed composition, animal breed, and age. Therefore, the effects of these factors on the bioactivity of steviol glycosides should be further explored in future studies.

### 4.3. Effect of Steviol Glycosides on Hu Sheep Rumen Fermentation

A comparison of the rumen pH between the two groups did not reveal any significant difference (*p* > 0.05). Rumen pH value is an important index reflecting the stability of the rumen fermentation environment, and its change may affect the community structure and activity of rumen microorganisms [24]. Although the change in pH in this study was small, future research needs to focus on its possible long-term effects on rumen health. In terms of NH_3_-N content, the addition of steviol glycosides significantly decreased the concentration of NH_3_-N (*p* < 0.05). The rumen concentration of NH_3_-N is the sum of rumen protein degradation and recycled nitrogen in the form of urea. Most of the nitrogen utilized by rumen microorganisms is in the form of NH_3_-N [25]. Low levels of rumen NH_3_-N reduce the digestibility of fiber because it is the only source of nitrogen for fiber-breaking microorganisms. Therefore, the relationship between low levels of NH_3_-N and low proportions of acetic acid in the rumen of steviol glycosides-consuming animals may impair the digestibility of dietary fiber. Last but not least, the high abundance of firmicutes and the low abundance of Bacteroidetes may indicate that there are fewer fiber-degrading bacteria in the rumen. This study found that the addition of steviol glycosides had no significant effect on the concentrations of PA, IBA, BA, IVA, and VA (*p* > 0.05), but AA was significantly decreased (*p* < 0.05). AA is the main precursor of milk fat synthesis in ruminants, and PA is an important precursor of glucose in ruminants. Therefore, PA fermentation can provide more energy for the body and is conducive to livestock fattening. Steviol glycosides may improve the energy utilization efficiency of Hu sheep by influencing rumen fermentation type [26]. In addition, some studies have also supported the positive effects of steviol glycosides on rumen fermentation. It has been reported that steviol glycosides can improve rumen environment feed digestibility and utilization rate [10]. It also has antibacterial and antioxidant effects, which help to maintain the ecological balance of rumen microorganisms and animal health [27]. In conclusion, the addition of steviol glycosides had a certain effect on the rumen fermentation parameters of Hu sheep but had no significant effect on production performance. I think the reasons for this result may be that steviol glycosides have no real effect on growth performance or that any potential effects are too small to be detected within the limitations of the experiment. It can also be caused by external environmental conditions, such as the time of the test or drinking water. Further tests are needed to confirm this.

### 4.4. Effect of Steviol Glycosides on Microbial Diversity of Hu Sheep

According to the results of Alpha diversity analysis, Shannon–Wiener, Simpson, Ace, and chao1 indexes of the CON were significantly higher than those of the STE, indicating that the rumen microbial diversity of the CON was more abundant. This may mean that steviol glycoside addition limited the growth of certain microorganisms or promoted excessive proliferation of specific microorganisms, thereby reducing overall diversity [28]. Similar studies have been reported in rumen microbes of pigs and cattle, and certain additives or changes in feed can affect the community structure and diversity of rumen microbes [29,30].

The dilution curve tends to be flat, indicating that the sequencing depth is reliable enough to truly reflect the composition of most microorganisms in samples, providing a solid basis for microbial diversity analysis [31]. Further analysis showed that although the two groups shared 792 OTUs, 3156 unique OTUs in STE and 1782 unique OTUs in CON, indicating that steviol glycosides addition significantly changed the community composition of rumen microorganisms. The introduction of new microbial species or the reduction of some existing microbial populations may affect the rumen fermentation process and nutrient utilization efficiency [32]. PCoA is a common method of visualization of Beta diversity, which can show the differences and similarities of microbial community structure between different samples [33]. PCoA analysis showed that there were differences in rumen microbial community structure between STE and CON, indicating that dietary steviol glycosides affect rumen microbial diversity and species composition of Hu sheep. This change may be caused by the fermentation of steviol glycosides in the rumen, which in turn affects the microbial community composition and diversity. Changes in microbial diversity may further affect rumen fermentation efficiency and nutrient absorption [34].

From the perspective of phylum level, *Firmicutes* and *Bacteroidetes* are the two most abundant groups of rumen microbial communities, which play a key role in maintaining rumen function and nutrient metabolism [35]. In this study, there was no significant difference in the relative abundance of *Firmicutes* and *Bacteroidetes* after the addition of steviol glycosides (*p* > 0.05). This suggests that steviol glycosides may not have a direct significant effect on microbes of the two groups or that the effect may take longer or larger doses of steviol glycosides to manifest itself [36]. In contrast, *Proteobacteria* increased significantly in STE (*p* < 0.05). This is because when the rumen microbiome is affected by external factors, certain members of *Proteobacteria* may increase [37]. In addition, *Patescibacteria* and *Desulfobacterota* also showed a significant downward trend after steviol glycosides addition (*p* < 0.05), which implies that steviol glycosides have some inhibitory effect on microorganisms of the two groups, but the mechanism of this inhibitory effect still needs to be further studied [38]. At the genus level, *Prevotella* is one of the most important genera in rumen, which is involved in carbohydrate degradation and energy metabolism [39]. The relative abundance of *Prevotella_7* and *Prevotella* decreased significantly under steviol glycosides treatment (*p* < 0.05). This may be due to the fact that steviol glycosides alter the supply of carbon sources in the rumen, resulting in weak competition for substrates among members of the genus *Prevotella* [40]. Another significant change was that the relative abundance of *Succinivibrionaceae_UCG_001* also increased significantly in STE (*p* > 0.05), which may be related to the metabolic pathway of steviol glycosides, as members of the genus *Succinivibrionaceae_UCG_001* are often able to use certain specific carbon sources for growth and metabolism [41]. The same result of *uncultured_rumen_bacterium* and *Rikenellaceae_RC9_gut_group* (*p* < 0.05). These changes may represent changes in some microbial groups that have not been fully studied under the influence of steviol glycosides [33]. The LDA score matrix gives us the difference in the relative abundance of different bacterial classes in the two samples. It can be clearly seen from the figure that after the addition of steviol glycosides, the LDA scores of some bacterial categories were higher, indicating that the relative abundance of these bacterial groups increased under the influence of steviol glycosides [42]. For example, *Gammaproteobacteria*, *Succinivibrionaceae*, and certain groups belonging to *Enterobacterales* scored higher in STE samples. These changes may mean that the addition of steviol glycosides promotes the growth or activity of these flora in the rumen of Hu sheep [43]. Among them, *Succinivibrionaceae* is a type of microorganism that can efficiently ferment to produce succinic acid. The change in its abundance in the rumen microbial community directly affects the fermentation pattern and metabolite generation in the rumen. With the increase in the number of *Succinivibrionaceae*, the fermentation environment in the rumen may be significantly improved, thereby enhancing the digestibility and utilization of feed [44]. The addition of steviol glycosides promoted the growth or activity of some beneficial flora while possibly inhibiting some others. These changes may have a positive impact on nutrient absorption and health of Hu sheep, but further studies are needed to explore their mechanisms of action and practical effects in depth [45]. The correlation analysis showed that AA was negatively correlated with *Succinivibrionaceae_UCG_001*, and IBA was positively correlated with *Lactobacillus*. *Succinivibrionaceae_UCG_001* is beneficial to the degradation of dietary fiber. The increase of *Succinivibrionaceae_UCG_001* results in a decrease in AA content. When A/P is low, it reflects the non-structural carbohydrate fermentation dominated by PA in the rumen. This fermentation pattern may affect energy utilization and milk fat synthesis in ruminants [46]. *Lactobacillus*, a probiotic that belongs to the phylum *Firmicutes*, affects the environment inside the rumen by producing lactic acid and other organic acids. The accumulation of lactic acid will reduce the pH of the rumen, and the decrease of rumen pH can inhibit the growth of some harmful microorganisms [47].

In conclusion, the addition of steviol glycosides significantly changed the diversity and community structure of rumen microorganisms in Hu-sheep. This change may have a profound impact on the rumen fermentation process, digestion, and absorption of nutrients. Future studies are needed to further explore the effects of these changes on the production performance of Hu sheep and optimize the amount and method of steviol glycosides supplementation to achieve the best feeding results.

## 5. Conclusions

The addition of steviol glycosides in the diet has no significant effects on the growth rate and serum biochemical indices of Hu sheep but can improve the rumen fermentation parameters and rumen microbial community structure, have a certain impact on the rumen microbial diversity and composition of sheep, and help maintain the rumen microecological balance. We suggest that farmers appropriately add steviol glycosides to regulate the rumen health of Hu sheep.

## Figures and Tables

**Table 1:** Composition and nutrient level of concentrate feed (DM basis).

Items	Content (%)	Nutrient Level	Content
Corn	65.80	DE/(MJ/kg)	13.81
Wheat bran	4.00	CP/%	16.62
Soybean meal	20.00	Ca/%	1.05
Flax cake	4.00	P/%	0.43
NaCl	0.60	NaCl/%	0.89
Baking soda	1.40		
Premix	4.00		
De-mold agent	0.20		
Total	100.00		

The premix provided the following per kg of the diet: Cu 5.40 mg, Fe 19.80 mg, Zn 9.00 mg, Mn 14.40 mg, Se 0.11 mg, I 0.18 mg, Co 0.07 mg, VA 7 200 IU, VD 1 440 IU, VE 500 IU.

**Table 2:** Effect of steviol glycosides on growth performance of Hu sheep.

Items	CON	STE	*p*-Value
Initial weight (kg)	17.63 ± 0.33	18.33 ± 0.33	0.157
Final weight (kg)	35.83 ± 1.45	36.88 ± 1.36	0.605
Net gain (kg)	18.20 ± 1.51	18.10 ± 1.45	0.962
ADG (kg)	0.20 ± 0.02	0.20 ± 0.16	0.962
ADFI (kg)	0.92 ± 0.05	0.98 ± 0.05	0.413
F/G	4.79 ± 0.54	4.90 ± 0.64	0.600

In the same row, values with no letter superscripts mean no significant difference (*p* > 0.05).

**Table 3:** Effect of steviol glycosides on serum biochemical indices of Hu sheep.

Items	CON	STE	*p*-Value
ALT (U∙L^−1^)	25.80 ± 2.56	24.74 ± 3.56	0.812
AST (U∙L^−1^)	130.53 ± 11.65	132.26 ± 15.31	0.930
TP (g∙L^−1^)	77.55 ± 2.01	74.81 ± 2.17	0.376
ALB (g∙L^−1^)	35.40 ± 0.83	35.31 ± 0.94	0.944
TG (mmol∙L^−1^)	0.32 ± 0.01	0.37 ± 0.07	0.486
CHO (mmol∙L^−1^)	1.43 ± 0.79	1.42 ± 0.11	0.968
HDL (mmol∙L^−1^)	0.81 ± 0.04	0.78 ± 0.65	0.632
LD (mmol∙L^−1^)	0.35 ± 0.26	0.38 ± 0.34	0.484
BUN (mmol∙L^−1^)	7.60 ± 0.60	8.41 ± 2.18	0.727
GLU (mmol∙L^−1^)	3.07 ± 0.25	2.99 ± 0.36	0.871

In the same row, values with no letter superscripts mean no significant difference (*p* > 0.05).

**Table 4:** Effect of steviol glycosides on rumen fermentation parameters of Hu sheep.

Items	CON	STE	*p*-Value
pH	6.77 ± 0.43	6.40 ± 0.44	0.247
NH_3_-N (mg∙100 mL^−1^)	7.21 ± 0.38 a	5.11 ± 0.51 b	0.002
Acetic acid (%)	52.18 ± 2.46 a	46.2 ± 3.98 b	0.011
Propionic acid (%)	36.76 ± 3.85	41.64 ± 5.78	0.116
Isobutyric acid (%)	0.4 ± 0.25	0.44 ± 0.13	0.767
Butyric acid (%)	7.8 ± 1.96	8.59 ± 2.26	0.531
Isovaleric acid (%)	1.09 ± 0.37	0.97 ± 0.26	0.519
Valeric acid (%)	1.77 ± 0.52	2.16 ± 0.72	0.305
TVFA (mmol∙L^−1^)	56.79 ± 18.37	61.8 ± 12.97	0.597
A/P	1.44 ± 0.22	1.14 ± 0.25	0.054

In the same row, values with different small letters mean a significant difference (*p* < 0.05), and with no letter superscripts mean no significant difference (*p* > 0.05).

**Table 5:** Comparison of the differences in Alpha diversity between the two groups.

Items	CON	STE	*p*-Value
Shannon–Wiener	7.9 ± 0.77 a	6.6 ± 0.69 b	0.012
Simpson	0.99 ± 0.01 a	0.97 ± 0.01 b	0.022
Chao1	968.12 ± 245.24 a	602.56 ± 243.20 b	0.027
Ace	975.21 ± 245.98 a	608.15 ± 244.60 b	0.027

In the same row, values with different small letters mean a significant difference (*p* < 0.05), and with no letter superscripts mean no significant difference (*p* > 0.05).

**Table 6:** Relative abundance of rumen microorganisms at gate level (%).

Items	CON	STE	*p*-Value
*Firmicutes*	53.861 ± 0.133	48.858 ± 0.059	0.428
*Bacteroidetes*	39.346 ± 0.098	32.353 ± 0.035	0.15
*Proteobacteria*	2.326 ± 0.029 b	17.096 ± 0.066 a	0.002
*Actinobacteria*	0.621 ± 0.003	0.951 ± 0.005	0.195
*Spirochaetota*	0.998 ± 0.011	0.094 ± 0.001	0.109
*Fibrobacteres*	0.996 ± 0.015	0.035 ± 0.001	0.174
*Patescibacteria*	0.515 ± 0.002 a	0.156 ± 0.002 b	0.013
unclassified_*Bacteria*	0.417 ± 0.003	0.214 ± 0.002	0.136
*Desulfobacterota*	0.433 ± 0.002 a	0.116 ± 0.001 b	0.004
*Verrucomicrobia*	0.233 ± 0.002	0.009 ± 0	0.066

In the same row, values with different small letters mean a significant difference (*p* < 0.05), and with no letter superscripts mean no significant difference (*p* > 0.05).

**Table 7:** Relative abundance of rumen microorganisms at genus level (%).

Items	CON	STE	*p*-Value
*Prevotella_7*	4.008 ± 0.089 b	19.036 ± 0.1 a	0.02
*Prevotella*	12.669 ± 0.059	7.028 ± 0.069	0.16
*Succinivibrionaceae_UCG_001*	1.714 ± 0.03 b	16.616 ± 0.064 a	0.001
*Selenomonas*	9.33 ± 0.075	4.784 ± 0.026	0.21
unclassified_*Selenomonadaceae*	2.077 ± 0.008	8.731 ± 0.064	0.052
*Succiniclasticum*	5.306 ± 0.021	4.776 ± 0.032	0.742
unclassified_*Lachnospiraceae*	4.281 ± 0.034	5.048 ± 0.043	0.739
uncultured_rumen_bacterium	7.081 ± 0.05 a	1.78 ± 0.023 b	0.04
*Shuttleworthia*	2.411 ± 0.049	4.538 ± 0.03	0.39
*Rikenellaceae*_*RC9*_gut_group	5.171 ± 0.029 a	0.757 ± 0.005 b	0.012
Others	45.902 ± 0.11 a	26.808 ± 0.063 b	0.004
Unclassified	0.05 ± 0	0.098 ± 0.002	0.483

In the same row, values with different small letters mean a significant difference (*p* < 0.05), and with no letter superscripts mean no significant difference (*p* > 0.05).

## Data Availability

The data presented in this study are available on request from the corresponding author.
